# Discordant experiences of food insecurity within households in Cameroon: an examination of data from the 2018 Demographic and Health Surveys

**DOI:** 10.1017/S1368980025100578

**Published:** 2025-06-17

**Authors:** Caroline E. Owens, Zhenan An, Craig Hadley

**Affiliations:** 1 Food is Medicine Institute, Friedman School of Nutrition Science and Policy, Tufts University, Boston, MA, USA; 2 Department of Quantitative Theory and Methods, Emory University, Atlanta, GA, USA; 3 Department of Anthropology, Emory University, Atlanta, GA, USA

**Keywords:** Food security, Gender, Wealth, Urbanicity, Households

## Abstract

**Objective:**

To assess the degree to which cohabiting couples (men and women) in Cameroon responded differently to the Food Insecurity Experience Scale and, where discordance exists, to test hypothesised drivers of difference.

**Design:**

This cross-sectional study employed descriptive statistics and multivariable regression analyses using R.

**Setting:**

Nationally representative sample of cohabiting adults in Cameroon.

**Participants:**

2889 couples (male/female; 5778 total adults) from the Cameroon Demographic and Health Survey (2018) couples recode.

**Results:**

Food insecurity was more prevalent and reported with higher severity among men compared with women. Discordance in reported food insecurity was evident in 57–79 % of cohabiting couples in the dataset, depending on the measure used. Discordance was not clearly associated with household wealth. Further, among couples with discordant food insecurity experiences, men more often affirmed items that their partners did not affirm. Contrary to our hypotheses, items reflecting *household* food security did not show greater agreement among couples than did individual items. Of our hypothesised predictors, only current employment status among men was significantly associated with the difference in food security scores among couples.

**Conclusions:**

This study highlights the importance of examining intrahousehold differences in food security. Understanding how individuals within a household experience and perceive their food situation and the underlying factors driving disparities is crucial for improving the effectiveness of targeted food and nutrition policies.

In 2023, an estimated 691–783 million people experienced food insecurity^(1)^. Defined as limited or uncertain access to nutritionally adequate and safe foods, food insecurity encompasses dimensions of availability, access, utilisation and stability. Globally, women bear a disproportionate burden of food insecurity^(2,3)^. Data from the Gallup World Polls indicate that 53 % of the world’s food insecure are women, who have a higher probability of reporting food insecurity relative to men, with variation across countries^(2)^. Social and environmental factors, such as racial and ethnic identity, wealth and rurality, also intersect with sex/gender to influence the risk of food insecurity. For example, rural women in lower- and middle-income countries are more likely than men and their urban counterparts to experience food insecurity and poor health^(4)^. Despite documented disparities, most research on food insecurity relies on household-level metrics, which extrapolate the food insecurity experience of one member, often an adult woman, to represent the entire household^(5)^. While widely applied, these metrics assume that all household members have equal physical and social access to food, overlooking research that documents distinct domains of responsibility related to food acquisition and provisioning. Recognising the limitations of household-level data, a growing number of food insecurity scales now assess individual-level experiences. Among these, the Food Insecurity Experience Scale (FIES), developed by the FAO, is one of the most widely employed^(6,7)^. The FIES examines experiential aspects of food insecurity and has documented cross-cultural relevance and validity. To identify individual-level differences within households, our study examines food insecurity among cohabiting couples in Cameroon, drawing on data from the FIES within the 2018 Demographic and Health Surveys (DHS).

Our study builds on a long-standing body of research exploring intrahousehold differences in resource allocation. Early studies on sex/gender-based disparities in food and nutrition emerged following observations of sex-skewed mortality, morbidity and malnutrition. Though studies frequently found that intrahousehold food distribution was sex-neutral after adjusting for body weight and activity patterns^(8,9)^, a pro-male bias was evident in several studies of children and youth in South Asia, reflecting a cultural preference for male offspring^(10,11)^. Notably, these studies from South Asia showed that intrahousehold inequities in food were not necessarily linked to poverty, as inequities did not decrease with income^(10)^. In contrast, greater disparities were evident among more educated and wealthier groups, which may have reflected the financial stress associated with marriage and dowry costs of daughters. A recent systematic review of food allocation between adults in South Asia confirms these findings, with the highest disparities in households experiencing severe or unexpected food insecurity and in better-off, higher-caste households^(11)^.

Research in several world regions has also shown that while overall food quantity is often allocated equitably within households, disparities are more pronounced in food quality, particularly in micronutrient intake. For example, Gittelsohn *et al.* found that women and girls in rural Nepal consumed fewer vitamin A and iron-rich foods than other household members due, in part, to culturally prescribed food rules for reproductive-aged females^(12)^. Similarly, research in Guatemala found a pro-male bias in protein allocation, favouring male heads of household^(13)^. Trade-offs between quantity and quality were also evident in studies in the Philippines, where preschoolers had nutritionally favourable diets but received greater portions of less preferred foods^(9)^. Collectively, these studies suggest that even when food *quantity* is equitable within the household, there may be disparities in food *quality and preferability*, which can drive perceptions of food insecurity.

Experiences with food insecurity are, therefore, driven by social and economic marginalisation and control and access to resources. However, as DeRose, Das and Millman emphasise, women’s limited autonomy in public spheres or lack of access to education and income does not necessarily correlate with power or control within households, especially in food-related matters^(14)^. In many contexts, women’s greater involvement with food acquisition and preparation may provide more access to food than is afforded to men. Alternatively, women’s relations with food may promulgate more sensitivity, worry or anxiety in response to food insecurity and the pressures of feeding a family. Men and women may also differentially prioritise food over other resources; for instance, Haddad *et al.* show that women are more likely than men to allocate income towards food, highlighting how control over income can alter intrahousehold food availability^(15)^. Cultural and ideological beliefs – such as perceptions that girls are less active or eat less or food rules that classify specific foods as taboo for certain subgroups – further influence intrahousehold access to food and nutrition^(9,16)^.

Research using experiential food insecurity scales, like the FIES, continues to reveal intrahousehold differences across sex/gender. For instance, in Ethiopia, Hadley *et al.* used an experiential food security scale among youth and showed that lower household wealth was associated with lower food security for girls but not for boys, although this differential disappeared when food prices rose^(17)^. Closer to our study, research in Bangladesh demonstrated discordance in food insecurity experiences among cohabiting adult men and women^(18)^. Specifically, Coates *et al.* observed that, when there is discordance, men were more likely to affirm questions about the augmentation of household resources in the public sphere (e.g. taking food on credit), whereas women were more likely to affirm questions about coping in a more private sphere (e.g. borrowing food from a neighbour), including behaviours like reduced consumption and reliance on less preferred or non-normative foods^(18)^. These findings show how sex/gender may influence food insecurity perceptions and responses within households, with clear ramifications for population-level estimates of insecurity and related health inequities. It is likely that other individual and household-level factors, including age, beliefs, attitudes towards gender equality and wealth, influence discordance between household members by shaping power dynamics and responsibilities; however, these factors have not been robustly examined in a nationally representative sample. Our analysis seeks to fill this gap by examining how wealth, rurality and indices of women’s empowerment influence sex/gender differences in food insecurity within households.

Data on food insecurity using the FIES from the Cameroon DHS enable us to explore these dynamics. Notably, the FIES has also been administered in Cameroon as part of the Gallup World Poll, which assesses factors related to world development indicators, including Sustainable Development Goals. In an analysis of this data, Broussard found that, in sub-Saharan Africa, gender inequality in food insecurity primarily occurred within severe food insecurity, in part due to the high rates of mild or moderate levels of food insecurity in the region^(2)^. Specifically, women were 2·7 percentage points more likely than men to experience severe food insecurity, which could be explained by gender differences in observed covariates (gender differences in household income, educational attainment and employment status)^(2)^. In contrast to regional findings, a recent analysis of data from the Cameroon DHS found that men are more likely to experience food insecurity than women^(19)^. However, to our knowledge, our analysis is the first to explore whether these disparities persist within cohabiting couples. Despite a rising economic growth rate of nearly 4 %, poverty levels in Cameroon have remained relatively stagnant, with an estimated 23 % of people living below the international poverty line of US $2·15 *per capita* per day in 2023^(20)^. These issues are exacerbated by conflict, particularly in the Northern region, and climate events such as droughts and floods that impact people engaged in the agricultural sector^(21)^.

Our study aimed to identify the extent of agreement among cohabiting mixed-sex couples regarding their individual food insecurity status. We examine whether both partners report the same level of food insecurity and, where discordance exists, investigate hypothesised drivers of discordance. More specifically, we hypothesise that intrahousehold sex/gender differences will occur such that women will report greater food insecurity and that these differences will be driven, in part, by decision-making autonomy, social independence and attitudes against gender-based intimate partner violence.

We relied on data from the 2018 DHS in Cameroon, which focused on couples, each of whom answered the eight-item FIES and provided data on a range of variables that potentially explained discordance. Based on previous research, we hypothesised that, in general, women would report food insecurity more than men; there would be intrahousehold discordance in reported severity of food insecurity; and that, in cases of discordance, women would be more likely to affirm alterations in food behaviour. We hypothesised that discordance would be lowest for item 6 of the FIES, ‘Your household ran out of food’, as the referent for this item is the household, whereas all other items refer to individual experiences. With respect to the degree of discordance, we hypothesised that the difference in men’s and women’s food security would be greatest in the poorest households, and we hypothesised that in households where the wife experienced lower empowerment, there would be a greater difference in the couple’s food security scores and higher likelihood of discordance classification.

## Methods

The data for this analysis come from the DHS (www.measuredhs.com). The DHS programme provides population-representative sample surveys on demographic and health-related topics from numerous lower and middle-income countries. We rely on the couples recode dataset from the DHS programme, which combines household and individual-level variables for couples. For Cameroon, this includes 2924 couples, though we removed 34 couples who were not actively living together because they are not reporting on the same household experience. Analysis was conducted using R^(22)^. This study was deemed non-human subject research by the Tufts University Institutional Review Board.

### Measuring food security and intrahousehold discordance

We use individual-level food security, as measured by the FIES, as our primary outcome variable. Our method closely follows the approach outlined in Coates *et al.* (2010, though they used the FANTA scale). For each respondent’s answers to the FIES, we used the FAO’s online tool (https://fies.shinyapps.io/ExtendedApp/) to generate item-fit statistics and to produce estimates of each respondent’s food security level: secure, moderately insecure or severely insecure. This analysis revealed that, for women, item 8 (going the whole day without eating) has an excessively higher outfit statistic (2·575). Our analyses demonstrated that the overall results were unaffected by the inclusion/exclusion of item 8; we therefore retained all FIES items for analyses. The degree and type of intrahousehold discordance (a case in which a female adult member responded affirmatively, and a male member did not and vice versa) were calculated for each item, along with population-level gender differences in responses to each item. Additionally, for each item in the FIES, we compared the prevalence of affirmative endorsement by men and women.

### Analysis

Our general analytic strategy for addressing hypotheses 1–4 was to compare mean levels of the FIES for men and women and within couples (hypothesis 1) and across strata of rural/urban place of residence (hypothesis 1) and household wealth (hypothesis 2). We cross-tabulated the food security categories of couples to assess the level and degree of discordance (hypothesis 3). For hypotheses 4 and 5, we calculated the sex/gender-specific prevalence of endorsement by item and used a *χ* ^2^ test to test for significant differences in item endorsement by gender.

To test hypothesis 6, we fit a multivariable linear regression where the outcome was the *difference* in food security score between members of the couple (woman’s food security score - man’s food security score). Positive values indicate that the female partner reported being *more* food insecure than her male partner. In these models, we control household wealth and the type of area of residence. To assess whether couples’ agreement on their food security status varied by household wealth, we used the DHS wealth index, which is generated through principal components analysis of household characteristics and ownership of material goods, land and animals. This provides an internally consistent wealth ranking within a country. The DHS produces a continuous wealth variable and a categorical variable labelled as poorest, poor, middle, richer and richest. Households are also categorised by the DHS as being in an urban or rural setting, and we rely on this classification for our analyses using the place of residence.

To operationalise gender-biased power dynamics within the household, we used the survey-based women’s empowerment index (SWPER), a measure of women’s empowerment for lower and middle-income countries^(23,24)^. The survey-based women’s empowerment index generates estimates of empowerment across three domains indicative of access to assets and agency: social independence, which primarily captures factors that enable women to achieve goals; decision-making, which captures women’s participation in household decisions; and attitudes to violence, which captures beliefs about gender norms-related to acceptability of intimate partner violence. On each of these factors, we hypothesised that as women scored lower, there would be a greater difference in food security scores and a higher likelihood of discordance. For details on the development and calculation of this index, we refer readers to Ewerling *et al.* 2020; in brief, the *attitude to violence* domain is composed of the following: Beating justified if wife goes out, if wife neglects children, if wife argues with husband, if wife refuses to have sex and if wife burns food. The *social independence* domain was made up of frequency of reading a newspaper or magazine, women’s education in completed years of schooling, age of woman at first birth (can be imputed for those who have not had a child), age at first cohabitation, age difference (woman’s minus husband’s age) and education difference (woman’s minus husband’s years of schooling), and the *decision-making* domain was comprised of items representing who decides on respondent’s health care, on large household purchases and on visits to family.

## Results

The final analytic sample included 2889 couples, of which 1287 were urban dwellers. The average age difference between couples (woman’s age – man’s age) was –8·3 years (sd 6·8), and the average difference in years of education within a couple (woman’s education – man’s education) was –1·3 (sd 3·5).

Hypothesis 1: Women would report food insecurity more than men, with a higher severity of reported food insecurity among rural women compared with urban women.

Women reported food insecurity scores that were consistently lower than men, regardless of location. Women reported an average food insecurity score of 3·5 (sd 3), while men reported an average of 4·6 (sd 2·9). Overall food insecurity scores were higher in rural areas compared with urban areas, but similar gender differences in food insecurity occurred in both rural and urban areas. Among rural dwellers, women reported an average food insecurity score of 4, while men reported 4·9; in urban areas, women reported mean scores of 3, and men reported mean scores of 4·2.

Hypothesis 2: The difference in men’s and women’s food security would be greatest in the poorest households.

Food insecurity scores were related to household wealth in a dose–response relationship for both men and women, but the *difference* in couples’ food insecurity scores was not systematically related to household wealth (Table 1). The Spearman’s correlation between household wealth and the difference in food insecurity scores among rural households was 0·035; in urban areas, it was 0·032; neither of these was statistically significant. Comparing the proportion of discordance, where households were assigned a one if there were differences in couples’ food insecurity scores and zero if there was agreement, across economic strata and location also revealed little in terms of a relationship between agreement and wealth (Table 2).

**Table 1.** Average difference in reported food insecurity among couples (woman’s score – man’s score) by wealth and place of residence

DHS wealth index	Urban	Rural
Poorest	−1·6	−1
Poorer	−1	−1·2
Middle	−1·4	−0·41
Richer	−1·3	−1·1
Richest	−1·2	−2·2

**Table 2.** Degree of discordance between couples’ food security assessments by wealth and place of residence

Wealth strata	Urban	Rural
Poorest	0·77	0·80
Poorer	0·81	0·79
Middle	0·82	0·80
Richer	0·80	0·79
Richest	0·74	0·72

Hypothesis 3: There would be intrahousehold discordance in the reported severity of food insecurity.

Next, we compared the categorical levels of food security as reported by each member of a couple to assess the degree of overlap, finding very low levels of concordance between couples living in the same households, as shown in Table 3. Overall, 56 % of couples were classified into different food security severity categories. Using the food security score instead of category, we found that 79 % of couples did not report the same food security score.

**Table 3.** Agreement between couples on their food security status (three-level classifications)

	Men’s level		
Women’s level	Secure	Moderate	Severe
Secure	24 %	14 %	14 %
Moderate	7 %	8 %	10 %
Severe	5 %	7 %	12 %

Hypothesis 4: Women would be more likely to affirm alterations in food behaviour.

Hypothesis 5: Discordance would be lowest for item 6, ‘Your household ran out of food’, as the referent for this item is the household, whereas all other items refer to individual experiences.

Counter to our hypothesis, our item-by-item analysis of the FIES items showed that men were more likely to affirm alterations in food behaviour (Table 4). Further, contrary to hypothesis 5, item 6, which referred to ‘the household’, did not have the lowest level of discordance; the highest levels of discordance were among the items ‘worried’, ‘skipped’ and ‘hungry’, although the levels of discordance across items were similar.

**Table 4.** Agreement and discordance between couples on individual Food Insecurity Experience Scale items

FIES item	Both disagree	Both agree	Wife (N), Hub (Y)	Wife (Y), Hub (N)	Discordance
Worried	20 %	38 %	26 %	17 %	42 %
Healthy	16 %	46 %	25 %	13 %	38 %
Few foods	18 %	41 %	29 %	12 %	41 %
Skipped	24 %	34 %	30 %	12 %	42 %
Ate less	22 %	37 %	28 %	13 %	41 %
Ran out	40 %	20 %	27 %	13 %	40 %
Hungry	38 %	20 %	28 %	14 %	42 %
Whole day	55 %	9 %	23 %	12 %	35 %

Hypothesis 6: In households where women reported lower decision-making autonomy, social independence and attitudes against gender-based intimate partner violence, discordance would be greater than in households in which women reported higher empowerment across these domains.

In the multivariable regression predicting the *difference* in the couple’s food security scores, there was no evidence of any statistically significant associations between our hypothesised predictor variables and the degree of difference in the couple’s assessments. In the ordinary least square model, shown in Table 5, wealth was not significantly associated with the difference in food security. None of the three measures of women’s autonomy and power were associated with the difference in food security. Specifically, the measure of the wife’s decision-making power was not associated with the difference in food security scores (Beta = 0·09, se 0·11), nor were the measures of attitudes towards spousal violence (0·08 se 0·09) or social independence (–0·10 se 0·11). The only variable that emerged as statistically significant was whether the man of the couple was employed; among couples where the man was employed the average difference in food security scores between men and women was –1·05, whereas among households where the man was not employed the average difference between couples’ food security scores was –1·85. The overall model had a very poor fit (F = 1·36, *P* = 0·20), and the r-square was effectively 0.

**Table 5.** Ordinary least squares (OLS) regression results for predicting intrahousehold differences in food security scores

Variable	Estimate	S e	*t*-Statistic	*P*-value
Intercept	−1·82	0·40	−4·56	<0·01
HH wealth	0·00	0·00	1·11	0·27
Rural	0·39	0·19	2·11	0·04
Decision-making	0·07	0·11	0·66	0·51
Social autonomy	−0·11	0·09	−1·28	0·20
IPV attitudes	0·07	0·09	0·85	0·40
HH size	0·00	0·02	−0·24	0·81
Male, employed	0·77	0·37	2·10	0·04
Female, employed	−0·26	0·16	−1·63	0·10

HH, Household; IPV, Intimate partner violence.

## Discussion

Our results, many of which do not support our initial hypotheses, reveal contextual nuances in the distribution of food insecurity across populations and within households. Overall, our results align with findings from other settings that co-residing men and women experience food insecurity differently, with discordance in reported experience evident in 56 % of couples in the Cameroonian DHS dataset. Coates *et al.* similarly identified discordance in reported food insecurity within 32 % of 576 households in Bangladesh, demonstrating intrahousehold variation in two distinct contexts^(18)^. These findings underscore that respondent identity matters for estimates of food insecurity, especially for household-level estimates that may be biased by gender-based reporting. For example, in our sample, if household-level prevalence estimates were extrapolated from individual-level data from female respondents, the experience of food insecurity from male respondents would be substantially underestimated. Notably, the UN Sustainable Development Goal 2, ‘End hunger, achieve food security and improved nutrition and promote sustainable agriculture’, relies on the FIES, an indicator for estimating moderate or severe food insecurity in the population.

While we document support for the hypothesis that intrahousehold discordance in food insecurity would occur, we did not find strong support for other hypotheses. In contrast to our primary hypothesis, in this dataset, the prevalence and severity of food insecurity were higher among adult men than adult women. Unexpectedly, our analysis revealed that adult men in this dataset experienced both a higher prevalence and greater severity of food insecurity compared with adult women. This finding contrasts with a robust body of literature documenting higher rates of food insecurity among women and girls^(2)^. Although societal norms around family provisioning impact men across the socio-economic spectrum, these pressures may be particularly acute in lower-income households, where limited resources challenge their ability to fulfil these roles. Food security exhibited a dose–response relationship with household wealth such that men and women in richer households were more food secure than those in poorer households. However, counter to our second hypothesis, the difference in couples’ food insecurity scores was not systematically related to household wealth. It is also conceivable that ongoing conflict in Cameroon contributes to a sense of precarity, which may disproportionately impact men. This could be tested further by examining regional differences in food insecurity while controlling for socio-economic confounders.

Further, our results do not support our hypothesis that women would be more likely to affirm alterations in food behaviour. Across each question in the FIES, men responded affirmatively more than their cohabiting female partners. Though Bishwajit and Yaya find this pattern in their analysis of DHS Cameroon 2018 data^(19)^, our results expand on their findings by demonstrating its persistence *within* households. Items on the FIES, including ‘you were unable to eat healthy and nutritious food’ and ‘you ate only a few kinds of foods’, foreground the social dimensions of insecurity; people may respond affirmatively to these items even if their caloric or dietary needs are met. Differences in how people conceive of ‘healthy, nutritious food’ and what constitutes ideal dietary variety may drive affirmative responses even in the context of what may be deemed food security based on caloric sufficiency alone. Unmeasured factors such as lack of desired or preferred foods may partially explain the higher reports of food insecurity among men in our sample. A number of studies have shown that people can consistently rank the desirability of foods^(25,26)^; if men are more concerned with the *quality* of their food intake, we would hypothesise that they would rate fallback foods as lower in preference. Studies that assess food preferences alongside validated measures of dietary intake, such as 24-hour dietary recalls or food frequency questionnaires, may reveal whether discrepancies between perceived dietary needs and actual intake drive reported food insecurity. Our study does not adjust for body weight or reported activity patterns, both of which may be higher among men, driving higher actual and perceived nutrient needs. Building on this idea, it is conceivable that women within the household feel their dietary needs are met at a lower nutrient quantity or quality threshold than men. Alternatively, as proposed by Bishwajit and Yaya, men’s higher food insecurity in Cameroon may reflect societal norms and expectations for men to provide for their families^(19)^.

Cohabiting women and men also evaluated their household’s food security differently, as demonstrated by the discordance between couples with item six of the FIES: ‘your household ran out of food’. Counter to our hypothesis, discordance on this item was not significantly lower than discordance on items that assess individual experiences. Our results differ from those of Coates *et al.*, who document that discordance among couples in Bangladesh was lower for items querying the household’s food situation^(18)^. This discrepancy in our study highlights that cohabiting individuals may have different understandings or perceptions of their household food situation in addition to differences in experience. Qualitative research with Hispanic adults and children in the USA highlights how familial roles, coping strategies, communication and interpretation shape intrahousehold discordance in food insecurity^(27)^. For instance, when communication or observation of food resources is limited, individuals less involved in food-related tasks may have a diminished awareness of their household’s food situation.

Finally, contrary to expectations, our proposed predictors of discordance – including household socio-economic traits (wealth, urbanicity), individual characteristics (employment) and indices of women’s empowerment (decision-making, social independence and attitudes towards intimate partner violence) – were not statistically significant and generally had small effects. Previous studies suggest that the generally disproportionate experience of food insecurity among women compared with men is driven, in part, by societal marginalisation and lack of power and autonomy within households. Findings from a recent systematic review suggest that proxies of women’s empowerment within households have a significant effect on household food security and that investment in women’s education and decision-making power are critical for advancing household food security^(28)^. In contrast, our findings suggest that these indicators are not significantly associated with *intrahousehold* differences in food security in this sample oif households from Cameroon. As other scholars have suggested, perceptions of empowerment are informed by local meanings and values and intersectional dimensions of identity. These indices are proxies for the structural determinants that shape food security rather than culturally attuned representations of sex/gender dynamics^(29)^. While our study does not investigate food insecurity across multiple intersections of identity, we do account for differences in livelihood and experience by including place of residence and household wealth in our analysis.

From a policy and public health perspective, these observed sex/gender differences suggest nuance in how progress towards development initiatives, such as ‘Zero Hunger’, is measured and evaluated. Our analysis suggests that, given the same levels of household food insecurity, the respondent’s gender may influence the reported severity of food insecurity. When respondents vary by gender, it is possible that the household’s food security level may vary depending on who in the household answered the questions. Further research could test this idea by assessing whether respondent gender identity predicts a household’s food security score, after controlling for wealth and sociodemographic characteristics.

Our reliance on proxy indices of women’s empowerment across domains of decision-making, social independence and attitudes towards intimate partner violence may not fully capture the complex dynamics of empowerment within households. Additionally, self-reported responses are subject to social desirability bias, which may influence accuracy. The cross-sectional nature of our data also fails to capture temporal variations in food security, which may further vary across livelihood, socio-economic status and sex/gender. Due to national security constraints, the 2018 DHS Cameroon dataset only includes thirteen of the intended forty-one clusters in the Northwest region and sixteen of forty in the Southwest, limiting national representation. Despite these limitations, our study is among the first to examine intrahousehold variation in food security within Cameroon using nationally representative data.

### Conclusions

This study reveals the importance of respondent identity, specifically sex/gender, in measuring food security, challenging assumptions embedded within household-level metrics. Future research could benefit from the development of context-specific metrics or the inclusion of qualitative approaches to better capture the factors driving intrahousehold food security disparities. Additionally, examining intrahousehold food security in diverse settings, including high-income countries, will be essential for understanding these dynamics globally. Such efforts are imperative for alleviating food insecurity, advancing key development aims and improving human well-being.
